# Gut microbiota variation in admixed Hispanic populations with polyendocrine metabolic ovarian syndrome

**DOI:** 10.3389/fendo.2026.1890094

**Published:** 2026-09-01

**Authors:** Loida A. Gonzalez-Rodriguez, Keishla M. Jimenez-Ortega, Daniela Vargas-Robles, Carolina I. Martínez-Hernández, Natalie M. Meléndez-Vázquez, Josefina Romaguera, Filipa Godoy-Vitorino

**Affiliations:** 1 Department of Internal Medicine, Endocrinology Division, School of Medicine, University of Puerto Rico Medical Sciences Campus, San Juan, Puerto Rico; 2 Department of Microbiology and Immunology, School of Medicine, University of Puerto Rico-Medical Sciences Campus, San Juan, Puerto Rico; 3 Department of Obstetrics and Gynecology, School of Medicine, University of Puerto Rico-Medical Sciences Campus, San Juan, Puerto Rico

**Keywords:** 16S rRNA sequencing, gut microbiome, hispanics, microbial diversity, polycystic ovary syndrome (PCOS), polyendocrine metabolic ovarian syndrome (PMOS)

## Abstract

**Introduction:**

Polyendocrine Metabolic Ovarian Syndrome (PMOS), referred to clinically as polycystic ovary syndrome (PCOS), is a common endocrine disorder associated with reproductive dysfunction, hyperandrogenism, insulin resistance, obesity, and chronic low-grade inflammation. Although gut microbiome alterations have been implicated in PMOS pathophysiology, data from Hispanic populations remain limited.

**Methods:**

This cross-sectional study evaluated fecal samples from 51 reproductive-age, non-menopausal women living in Puerto Rico, including 41 with PMOS, diagnosed following the Rotterdam criteria, and 10 healthy controls. Genomic DNA was obtained from fecal samples and subjected to 16S rRNA gene sequencing for bacterial identification. Microbial assessment included associations with metabolic, androgenic, and reproductive variables.

**Results:**

After adjustment for age, body mass index (BMI), and recent antibiotic use, PMOS status was not significantly associated with alpha nor beta diversity (p>0.05). However, host factors such as BMI and age explained more variation in microbial communities than PMOS status. Indicator species analysis identified several taxa preferentially associated with controls, including *Phocaeicola_A dorei*, *Alitiscatomonas aceti*, *Brotaphodocola*, and *Schaedlerella*. Specifically, *Fusicatenibacter saccharivorans* was significantly reduced in the PMOS group. Metabolic markers, androgen levels, and reproductive features were not significantly associated with global microbiome diversity or composition; however, irregular menses was associated with greater microbiome dispersion.

**Discussion:**

These findings suggest that PMOS is not characterized by major gut microbiome shifts but may involve subtle depletion of health-associated commensals and increased microbial heterogeneity.

## 1 Introduction

Polyendocrine Metabolic Ovarian Syndrome (PMOS), clinically recognized as polycystic ovary syndrome (PCOS), is one of the most common endocrinopathies affecting reproductive-aged women. It is characterized by hyperandrogenism, ovulatory dysfunction, and polycystic ovarian morphology (1). In addition to reproductive manifestations, PMOS is strongly associated with insulin resistance, obesity, chronic low-grade inflammation, and increased cardiometabolic risk (2). In this manuscript, the term PMOS is used to emphasize the multisystem metabolic and endocrine manifestations of the syndrome while acknowledging that PCOS remains the standard clinical nomenclature.

While genetic predisposition contributes to disease susceptibility, it does not fully account for the clinical presentation and disease progression. Admixed Hispanic populations, including those living in Puerto Rico, have a self-reported prevalence of PMOS of approximately 12.3% (3).

The gut microbiota, composed of bacteria, viruses, and fungi, plays a fundamental role in host metabolism, immune regulation, and endocrine signaling (4). Emerging evidence suggests that the gut microbiome modulates disease states such as obesity (5), insulin resistance (6), cardiovascular disease (7), and mental health (8). The gut microbiome may influence metabolic, hormonal, and inflammatory pathways associated with PMOS. Proposed mechanisms include microbial metabolites such as short-chain fatty acids (SCFAs), bile acids, and lipopolysaccharides (LPS), as well as immune and endocrine signaling pathways (9, 10). These findings position the microbiota–gut–ovary axis as a critical and potentially reversible mechanism underlying PMOS. In PMOS, gut dysbiosis has been increasingly recognized as a contributor to insulin resistance and hyperandrogenism (11). These pathways influence glucose homeostasis, lipid metabolism, and ovarian function. Disruption of balance can lead to increased intestinal permeability (“leaky gut”), systemic inflammation, and exacerbation of metabolic and hormonal disturbances characteristic of PMOS (12).

However, many of these associations remain correlative, and causal mechanisms have not been fully established.

Studies in diverse populations have identified specific microbial patterns associated with PMOS. A consistent finding is reduced microbial diversity and a relative decrease in beneficial SCFA-producing bacteria, particularly within the Firmicutes phylum (10). In addition, an increased abundance of potentially proinflammatory and opportunistic bacteria has been observed (10). This dysbiotic profile contributes to increased endotoxin production, such as LPS, which triggers systemic inflammation and worsens insulin resistance by activating inflammatory signaling pathways (10). Alterations in bile acid metabolism and reduced SCFA production impair glucose regulation, lipid metabolism, and satiety signaling. These changes can further exacerbate hyperinsulinemia, which in turn stimulates ovarian androgen production, creating a vicious cycle that perpetuates PMOS pathophysiology.

Dietary patterns are known to influence gut microbiome composition and metabolic health (13). Diets rich in refined carbohydrates and processed foods may worsen insulin resistance and inflammation, whereas fiber-rich foods, healthy fats, and minimally processed dietary patterns may support metabolic regulation and microbial diversity (14, 15). Puerto Rican populations have been reported to consume relatively low amounts of fruits, vegetables, and dietary fiber, which may indirectly influence microbiome composition and metabolic outcomes (16, 17). Although dietary intake was not directly evaluated in the present study, these factors may contribute to inter-individual microbiome variability. This is particularly relevant because low-fiber diets may adversely affect gut microbial diversity and contribute to insulin resistance, systemic inflammation, and metabolic dysfunction, which are key features commonly associated with PMOS. Furthermore, dietary patterns characterized by high consumption of processed foods and refined carbohydrates, alongside low intake of fiber-rich foods such as fruits, vegetables, legumes, and whole grains, may indirectly influence both gut microbial composition and the severity of PMOS-related symptoms. We hypothesize that women with PMOS will exhibit reduced microbial diversity and an increased abundance of pro-inflammatory taxa, and that these alterations will correlate with key clinical features such as insulin resistance and hyperandrogenism. Our findings provide novel insights into the gut microbiome of Puerto Rican women with PMOS and highlight potential microbiome-targeted therapeutic strategies to improve clinical outcomes in this population.

## 2 Materials and methods

### 2.1 Patient recruitment and sampling

This cross-sectional study evaluated the gut microbiome in premenopausal women with PMOS living in Puerto Rico. Participants were recruited from the ongoing study *Polycystic Ovary Syndrome and Metabolic Syndrome in a Hispanic Population*, which examines the association between PMOS and metabolic syndrome in women residing in Puerto Rico. Inclusion criteria included: (1) females aged 21–45 years presenting with at least one of the following: clinical hyperandrogenism (acne, alopecia, or hirsutism), overweight or obesity, and/or menstrual irregularities; (2) provision of written informed consent; and (3) willingness to comply with all study procedures and availability for study participation. Exclusion criteria included: (1) history of hysterectomy or bilateral oophorectomy; (2) history of uterine artery embolization or endometrial ablation; (3) pregnancy or breastfeeding; and (4) unwillingness to participate or inability to understand the consent process, study objectives, or study requirements. Recruitment was conducted in compliance with the Human Subjects Protection Office and approved by the Institutional Review Board of the University of Puerto Rico-Medical Sciences Campus (Protocols #2290034657 and #2290030271).

The sub-cohort study population consisted of 51 patients (41 with PMOS and 10 healthy controls). PMOS was diagnosed according to the Rotterdam Criteria (18), after exclusion of thyroid dysfunction, elevated prolactin levels, and non-classic congenital adrenal hyperplasia. After providing written informed consent, participants were scheduled for evaluation during the follicular phase of their subsequent menstrual cycle (days 1–10). Each participant underwent a comprehensive clinical evaluation, including a detailed medical and gynecologic history, a complete physical examination with blood pressure measurements, anthropometric assessments (height, weight, waist circumference, and hip circumference), a dermatologic evaluation for hirsutism, acne, alopecia, and acanthosis nigricans, pelvic and breast examinations, and transvaginal and/or pelvic ultrasonography.

During dermatologic evaluation, standardized acne lesion assessment was used and scored as grade 1 (mild), grade 2 (moderate), or grade 3 (severe) (19). The Ludwig Scale was used to assess androgenic alopecia (20). Participants completed validated questionnaires, including the Patient Health Questionnaire-9 (PHQ-9), the 12-item Short Form Health Survey (SF-12), and a dietary patterns questionnaire, however the dietary pattern diet was not included in the analyses and we did not record frequency of low-fiber/high-refined-carb consumption, which could impact the gut microbiome. Following these assessments, participants were provided with standardized instructions for stool sample collection. Samples were collected by the participant at their home, stored at 4 °C, and transported within 24 hours to the Hispanic Alliance for Clinical and Translational Research facilities at the University of Puerto Rico-Medical Sciences Campus, where they were stored at −80 °C until analysis. No defecation status or timing relative to sample collection were noted.

### 2.2 Blood collection and laboratory assessments

Fasting blood samples were collected in the morning after a 12-hour overnight fast. Laboratory analyses, measured using the cobas 8000 Electrochemiluminescence Immunoassay (ECLIA) platform, included fasting glucose, fasting insulin, total and free testosterone, sex hormone-binding globulin (SHBG), dehydroepiandrosterone sulfate (DHEAS), thyroid-stimulating hormone (TSH), prolactin, lipid profile, C-reactive protein (CRP), and glycated hemoglobin (HbA1c) levels. To exclude non-classic congenital adrenal hyperplasia, 17-hydroxyprogesterone (17-OHP) levels were measured at Mayo Clinic Laboratories (Rochester, MN, USA). In addition, a 75-gram oral glucose tolerance test (OGTT) was also performed.

Clinical hyperandrogenism was evaluated using a modified Ferriman–Gallwey (mFG) score ≥8, regardless of the presence or absence of acne or alopecia (21, 22). Biochemical hyperandrogenism was defined as elevated total testosterone, free testosterone, DHEAS, or an increased free androgen index (FAI) above the laboratory-specific reference ranges. Oligo-/anovulation (OA) or menstrual dysfunction (MD) was defined as a menstrual cycle length of ≥ 35 days or ≤ 21 days, or the occurrence of ≤ 9 episodes of vaginal bleeding within a 12-month period.

Metabolic syndrome was defined according to the Adult Treatment Panel III (ATP III) criteria, requiring the presence of at least three of the following five components: (1) fasting plasma glucose ≥100 mg/dL (5.6 mmol/L) or treatment for elevated glucose levels; (2) high-density lipoprotein cholesterol (HDL-C) <50 mg/dL (1.3 mmol/L) or treatment for low HDL-C; (3) triglyceride levels ≥150 mg/dL (1.7 mmol/L) or treatment for hypertriglyceridemia; (4) waist circumference ≥88 cm (35 inches); and (5) blood pressure ≥130/85 mmHg or antihypertensive treatment. Insulin resistance was evaluated using the Homeostatic Model Assessment for Insulin Resistance (HOMA-IR), with a value ≥2.5 indicating insulin resistance (23, 24).

### 2.3 Ultrasound assessment

Transvaginal and/or pelvic ultrasonography was performed using a Voluson E8 Expert BT12 ultrasound system (GE Healthcare, Chicago, IL, USA). Sexually active participants underwent transvaginal ultrasonography using a GE R1C 5–9-D endocavitary transducer, whereas transabdominal ultrasonography was performed in participants who had never been sexually active using a RAM 3–8–12 M6C 3D/4D probe. Ultrasonographic evaluation included assessment of uterine dimensions and abnormalities, endometrial thickness, ovarian dimensions in three planes, largest follicular diameter, follicle count in the largest ovarian plane, and overall ovarian morphology. Polycystic ovarian morphology (PCOM) was defined as the presence of ≥12 antral follicles measuring 2–9 mm in diameter and/or an ovarian volume ≥10 cm³ in at least one ovary, in the absence of a dominant follicle or ovarian cyst.

### 2.4 Stool sample microbial analysis

#### 2.4.1 Genomic DNA and 16S rRNA sequencing

Genomic DNA (gDNA) was extracted from PMOS and non-PMOS fecal samples using the QIAGEN DNeasy PowerSoil Pro Kit (QIAGEN LLC, Germantown Road, Maryland, USA) according to the manufacturer’s protocol, with the following modifications: (1) 200mg of fecal sample was used, (2) vortexing was set at a maximum speed for 10 minutes followed by high-frequency disruption using a QIAGEN TissueLyser II (QIAGEN LLC, Germantown Road, Maryland, USA) for 3 minutes at a frequency of 30.0 (1/s), (3) centrifugation steps were performed at 13,500 rpm, and (4) elution was done with 75 μL of warmed (55-65 °C) C6 solution. gDNA quantifications were performed using the Qubit 1X dsDNA HS Assay Kit (high sensitivity; Thermo Fisher, Waltham, MA, USA) and the Qubit 2.0 Fluorometer. As described by the Earth Microbiome Project, the V4 hypervariable region of the 16S ribosomal RNA gene was amplified with the universal primers 515F (5’-GTGCCAGCMGCCGCGGTAA-3’) and 806R (5’-GGACTACHVGGGTWTCTAAT-3’). Bacterial amplicons were sequenced on an Illumina MiSeq using a 2 x 250 base paired-end protocol. The demultiplexed sequences were deposited in Qiita (25) and analyzed in RStudio (http://www.R-project.org) using a DADA2 denoising workflow (26). Subsequent script-based filtering steps removed mitochondria- and chloroplast-associated amplicon sequence variants (ASVs) prior to downstream analyses. Taxonomic assignment for ASVs was performed with the Greengenes2 reference database. DNA extraction blanks were processed alongside the samples as negative controls. These blanks yielded very few sequencing reads and were therefore excluded from the downstream analyses.

#### 2.4.2 16S rRNA sequencing analyses

Samples were rarefied to 9,015 sequences per sample prior to diversity analyses. Sequencing depth adequacy was evaluated using Good’s coverage index, computed with the *QsRutils* package (27). Coverage values were consistently high across the dataset, with median Good’s coverage reaching 100% before and after rarefaction, and with means of 100% (non-rarefied) and 96.8% (rarefied), indicating that the sequencing effort sufficiently captured the within-sample diversity. Microbiome diversity metrics were calculated using functions from the *vegan* (28) and *phyloseq* (29) packages.

#### 2.4.3 Alpha diversity metrics

Alpha diversity was quantified using Shannon, Simpson, and observed richness indices calculated from rarefied ASV tables. Associations with PMOS status were evaluated using multivariable linear regression models, with PMOS status as the main exposure and adjusted for age and body mass index (BMI). Where Shapiro–Wilk indicated departures from normality, results were corroborated using rank-based (Spearman) or Wilcoxon rank-sum comparisons; conclusions were consistent across approaches, and linear regression estimates are reported for ease of interpretation of covariate-adjusted effects. To account for potential effects of recent antibiotic exposure, analyses were performed both including antibiotic use within the prior month as a covariate and, in sensitivity analyses, excluding participants with recent antibiotic use. Visualizations were performed using scatter plots with group-specific regression lines.

#### 2.4.4 Beta diversity and clustering analysis

Differences in overall gut microbiome composition between PMOS and control participants were assessed using Bray–Curtis, Jaccard, and Aitchison (30) distances. Group-level differences were tested using permutational multivariate analysis of variance (PERMANOVA) with 9,999 permutations, adjusting for age and body mass index (BMI). Homogeneity of dispersion was evaluated using *betadisper* to confirm that PERMANOVA results reflected differences in community composition rather than within-group variability. Ordinations were visualized using non-metric multidimensional scaling (NMDS). Sensitivity analyses were conducted excluding participants with recent antibiotic use.

To further explore community-level structure independent of PMOS status, unsupervised hierarchical clustering was performed based on Bray–Curtis dissimilarity. Clustering was conducted using Ward’s minimum variance method (ward.D2) applied to a Euclidean representation of the Bray–Curtis distance obtained via principal coordinates analysis (PCoA). The optimal number of clusters was evaluated using average silhouette width. Because silhouette values were comparable across solutions, a two-cluster solution was selected to maximize interpretability and statistical power. Cluster membership was subsequently compared between PMOS and control participants using Fisher’s exact test, and clustering results were visualized using dendrograms and PCoA plots.

Associations between gut microbiome composition and metabolic markers were assessed using PERMANOVA (adonis2) based on Bray–Curtis, Jaccard, and Aitchison distances. Each metabolic marker (HOMA-IR, fasting insulin, HbA1c, and waist circumference) was evaluated in separate models while adjusting for age, BMI, and PMOS status. Statistical significance was assessed using 9,999 permutations with marginal tests. Results were summarized using R² values to compare the relative contribution of metabolic factors to overall microbiome variation.

#### 2.4.5 Discriminant taxa analysis

Differential abundance analyses were conducted using MaAsLin2 (31) across multiple taxonomic levels. Taxa were filtered based on prevalence and abundance thresholds prior to modeling. Models included PMOS status as the primary variable of interest and were adjusted for age and BMI. Primary analyses excluded participants with recent antibiotic use, with secondary models including antibiotic use as a covariate. Multiple testing was addressed using the Benjamini–Hochberg False Discovery Rate (FDR) procedure (q). Significance was defined as p < 0.050 and q <0.250.

#### 2.4.6 Correlations with metabolic markers

Associations between gut microbial alpha diversity and metabolic markers were assessed using Spearman’s rank correlation, given the non-normal distribution of several metabolic variables. Alpha diversity metrics included Shannon, Simpson, and observed richness indices. Metabolic markers evaluated were HOMA-IR, fasting insulin, HbA1c, and BMI. Correlations were computed using complete-case analysis, where p-values were adjusted for multiple testing using the Benjamini–Hochberg FDR procedure, and results were visualized using heatmaps.

#### 2.4.7 Circulating androgen levels

Associations between gut microbiome alpha diversity and circulating androgen levels were assessed using Spearman’s rank correlation. Alpha diversity metrics included the Shannon, Simpson, and observed ASV indices, all calculated from rarefied ASV tables. Androgen-related variables comprised total testosterone, FAI, and SHBG. Spearman’s correlation was selected due to the non-normal distribution of several variables. Analyses were conducted using pairwise complete observations. P-values were adjusted for multiple comparisons using the Benjamini–Hochberg FDR, and both raw p-values and q-values are reported.

Associations between overall gut microbiome composition and circulating androgen levels were evaluated using beta diversity analyses. Bray–Curtis, Jaccard, and Aitchison (centered log-ratio–transformed Euclidean) distance matrices were computed from rarefied ASV tables. For each distance metric, PERMANOVA was performed with 9,999 permutations to test associations with individual androgen-related variables, including total testosterone, FAI, and SHBG. Models were adjusted for age, BMI, and PMOS status to account for potential confounding. To ensure that PERMANOVA results were not driven by differences in within-group variability, homogeneity of dispersion was assessed using *betadisper*. Sample ordination was visualized using an NMDS, with samples colored by androgen levels for descriptive purposes.

#### 2.4.8 Menstrual and reproductive features

Associations between menstrual and reproductive characteristics and gut microbiome composition were evaluated using beta-diversity analyses. Menstrual variables included irregular menses, current oligomenorrhea, current amenorrhea, difficulty conceiving, and menstrual cycle length. Beta diversity was assessed using Bray–Curtis, Jaccard, and Aitchison (centered log-ratio–transformed Euclidean) distance metrics. For each menstrual or reproductive variable, PERMANOVA was performed with 9,999 permutations, adjusting for age, BMI, and PMOS status. Homogeneity of group dispersions was evaluated using *betadisper* to assess differences in within-group variability. Where dispersion differences were significant, results were visualized using boxplots of distances to group centroids for each category and distance metric.

## 3 Results

### 3.1 Population demographic and clinical characteristics

The study cohort consisted of 51 women, divided into a PMOS group (n=41) and a healthy control group (n=10). No significant differences were observed between groups for age or metabolic markers, including BMI, fasting glucose, fasting insulin, glycated hemoglobin (HbA1c), and HOMA-IR. Significant differences were identified in reproductive history, with the control group exhibiting more pregnancies (2.90 vs. 0.59, p<0.001), more abortions (1.20 vs. 0.12, p<0.001), and higher parity (1.70 vs. 0.46, p=0.002). Clinical manifestations of PMOS were evident, as the PMOS group had a significantly higher total modified Ferriman-Gallwey score (5.32 vs. 0.20, p=0.019) and a significantly higher prevalence of current oligomenorrhea (70.7% vs. 30.0%, p=0.043), alongside a near-significant trend for current amenorrhea (p=0.067). Regarding androgenic markers, while free testosterone, DHEAS, and SHBG showed no significant differences, marginal differences were observed for total testosterone, which was higher in PMOS (0.55 vs. 0.35, p=0.080) and FAI (6.36 vs. 3.45, p=0.064). Other menstrual and reproductive features relevant to the study, such as history of irregular menses, difficulty getting pregnant, current regular periods, and abnormal menstrual length, did not differ significantly between the groups (**Table 1**; **Supplementary Table 1**).

**Table 1:** Demographic and clinical variables of the study population including controls and PMOS participants.

Clinical variable	Overall (n=51)	Control (n=10)	PMOS (n=41)	p-value
Patient characteristics (mean ± SD)				
Age	33.27 ± 7.14	36.62 ± 8.27	32.46 ± 6.70	0.099
Weight (lbs)	197.97 (45.95)	194.49 (46.58)	198.82 (46.33)	0.792
Height (in)	64.20 (2.63)	63.68 (2.73)	64.33 (2.62)	0.489
BMI (mean ± SD)	33.72 (7.34)	33.72 (8.16)	33.72 (7.24)	1
Waist circumference (cm)	99.45 (17.70)	100.27 (19.09)	99.24 (17.59)	0.871
Hip circumference (cm)	117.08 (14.30)	116.94 (14.67)	117.12 (14.40)	0.972
Medical history n (%)				
Metabolic Syndrome	2 (3.9)	0 (0.0)	2 (4.9)	1
Amenorrhea	2 (3.9)	0 (0.0)	2 (4.9)	1
Irregular Menses	6 (11.8)	2 (20.0)	4 (9.8)	0.723
PMOS*	25 (49.0)	1 (10.0)	24 (58.5)	0.016
Other	34 (66.7)	9 (90.0)	25 (61.0)	0.17
Family history of PMOS	3 (5.9)	0 (0.0)	3 (7.3)	0.895
Medications n (%)				
Insulin	1 (2.0)	0 (0.0)	1 (2.4)	1
GLP-1 agonists	2 (3.9)	1 (10.0)	1 (2.4)	0.845
Metformin	12 (23.5)	2 (20.0)	10 (24.4)	1
Antibiotic Use				0.605
None in the past year	23 (45.1)	5 (50.0)	18 (43.9)	
During the past week	3 (5.9)	0 (0.0)	3 (7.3)	
During the past month	9 (17.6)	1 (10.0)	8 (19.5)	
Six months ago	9 (17.6)	3 (30.0)	6 (14.6)	
One year ago	4 (7.8)	0 (0.0)	4 (9.8)	
Not Available	3 (5.9)	1 (10.0)	2 (4.9)	
Gynecologic and obstetric history				
Pregnancies (mean ± SD)	1.04 (1.54)	2.90 (2.13)	0.59 (0.92)	<0.001
Abortions (mean ± SD)	0.33 (0.71)	1.20 (1.03)	0.12 (0.40)	<0.001
Parity (mean ± SD)	0.71 (1.17)	1.70 (1.70)	0.46 (0.87)	0.002
Duration of menstrual cycle (mean ± SD)	5.30 (2.54)	6.20 (4.49)	5.00 (1.60)	0.374
Regular menstrual cycle, n (%)	15 (29.4)	5 (50.0)	10 (24.4)	0.228
Irregular menstrual cycle, n (%)	37 (72.5)	5 (50.0)	32 (78.0)	0.165
Menorrhagia, n (%)	23 (45.1)	5 (50.0)	18 (43.9)	1
Metrorrhagia, n (%)	27 (52.9)	5 (50.0)	22 (53.7)	1
Oligomenorrhea, n (%)	32 (62.7)	3 (30.0)	29 (70.7)	0.043
Amenorrhea, n (%)	26 (51.0)	2 (20.0)	24 (58.5)	0.067
Difficulty getting pregnant, n (%)	20 (39.2)	2 (20.0)	18 (43.9)	0.304
Laboratory parameters (mean ± SD)				
Fasting glucose (mg/dL)	100.88 (16.24)	102.50 (6.80)	100.47 (17.88)	0.728
Fasting Insulin (µIU/mL)	17.01 (15.63)	12.30 (6.17)	18.16 (17.02)	0.292
Hemoglobin A1c (%)	5.49 (0.50)	5.45 (0.33)	5.50 (0.54)	0.805
Sex Hormone Binding Globulin (nmol/L)	41.41 (31.69)	42.91 (23.18)	41.04 (33.67)	0.869
DHEA sulfate (µg/dL)	182.81 (114.59)	158.59 (158.61)	188.71 (102.82)	0.462
Free testosterone (ng/mL)	0.02 (0.11)	0.01 (0.00)	0.03 (0.12)	0.544
Total testosterone (ng/mL)	0.51 (0.32)	0.35 (0.30)	0.55 (0.32)	0.08
HOMA-IR	4.58 (5.53)	3.16 (1.81)	4.93 (6.08)	0.371
Free Androgen Index	5.79 (4.47)	3.45 (3.30)	6.36 (4.56)	0.064

*The ‘PMOS’ medical history variable reflects prior self-reported diagnosis before study enrollment and is distinct from study-based Rotterdam classification Rotterdam Criteria and the modified Ferriman-Gallwey score were used.

### 3.2 Gut microbial diversity and composition between PMOS and control patients

**Figure 1:**
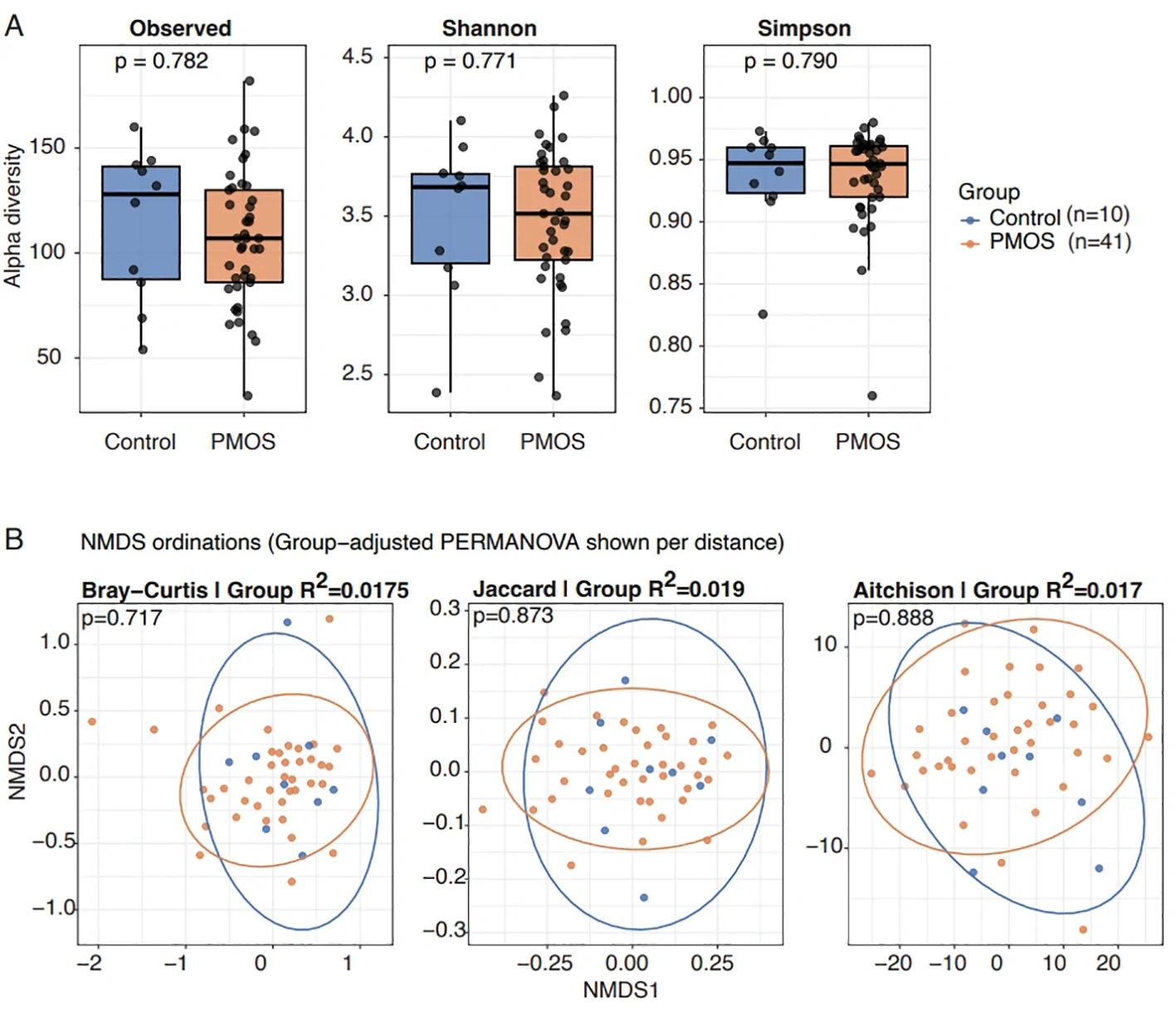
Gut microbial alpha and beta diversity in women with PMOS and controls. **(A)** Alpha diversity metrics (Observed, Shannon, Simpson) did not differ between groups in multivariable models adjusted for age, BMI, and recent antibiotic use. **(B)** Overall community composition did not differ between groups across Bray–Curtis, Jaccard, or Aitchison distances in PERMANOVA models adjusted for the same covariates.

Gut microbial diversity and overall community structure were comparable between women with PMOS and controls. In multivariable linear regression models adjusting for age, BMI, and recent antibiotic use, PMOS status was not associated with observed richness (p=0.782), Shannon diversity (p=0.771), or Simpson diversity (p=0.790, **Figure 1A**). Covariates, including age, BMI, and recent antibiotic exposure, were also not significantly associated with alpha diversity metrics. Similarly, overall community composition did not differ between groups. PERMANOVA models adjusted for the same covariates revealed no significant association between PMOS status and microbiome structure across Aitchison (p=0.888), Bray–Curtis (p=0.716), or Jaccard (p=0.872) distances (**Figure 1B**). These findings remained consistent in sensitivity analyses for alpha and beta diversity, excluding participants with recent antibiotic use (n=36; **Supplementary Tables 2**, **3**).

Host factors, such as BMI and age, explained a larger proportion of microbial variation than PMOS status alone. In the Aitchison distance model, BMI accounted for approximately 2.6% of the variance (R^2^ = 0.026), while PMOS status explained only 1.7% (R^2^ = 0.017). Similarly, in the Bray-Curtis model, BMI (R^2^ = 0.025) and age (R^2^ = 0.023) were stronger contributors than clinical group membership (R^2^ = 0.017). Recent antibiotic use contributed modestly to the variance and was not a significant driver of community structure in any model (p>0.26, **Supplementary Table 3**).

**Figure 2:**
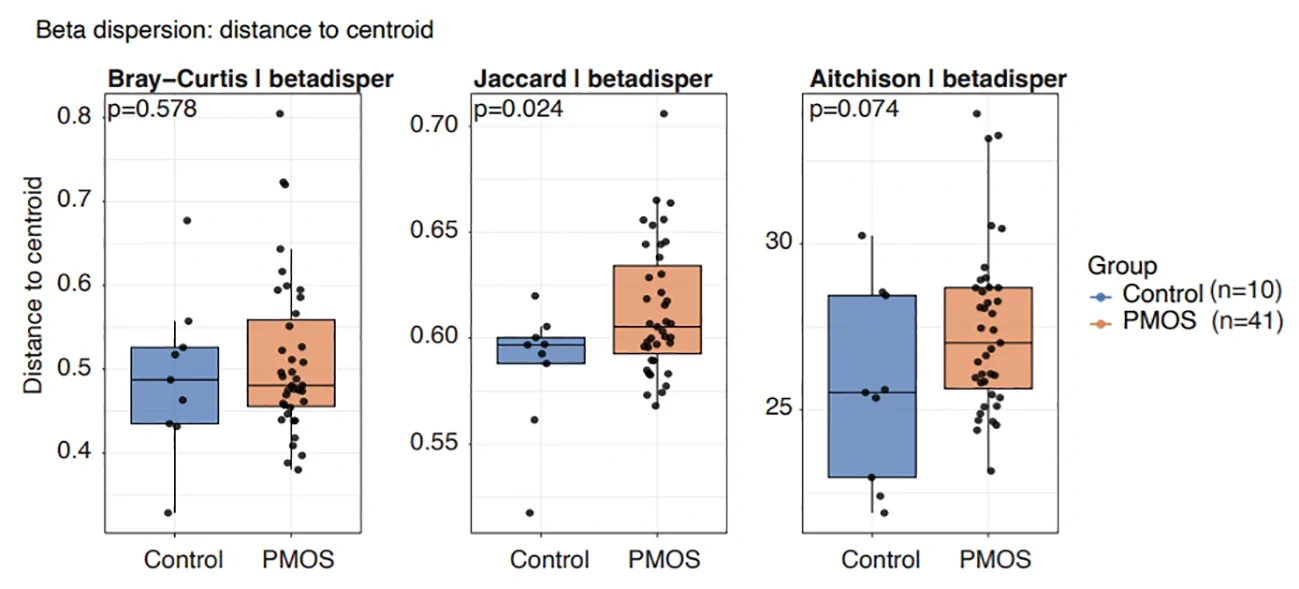
Gut microbial beta diversity dispersion in women with PMOS and controls. Distance to centroid (*betadisper*) was calculated for Bray–Curtis, Jaccard, and Aitchison distances.

To further assess whether group differences might be reflected in variability rather than centroid location, we next evaluated the homogeneity of multivariate dispersion. Tests for homogeneity of multivariate dispersion (*betadisper*) showed no significant differences in within-group variability for Aitchison (p=0.075) or Bray-Curtis (p=0.568) distances. However, a significant difference in dispersion was identified using Jaccard distances (p=0.022, **Figure 2**), indicating that the variance in microbial presence/absence patterns differs between groups. This significant difference in Jaccard dispersion persisted even after excluding participants who used antibiotics (p=0.032, **Supplementary Table 2**).

Finally, given that dispersion differences were restricted to presence/absence patterns, we examined whether unsupervised hierarchical clustering could identify community structures associated with PMOS status. Clustering based on Bray–Curtis dissimilarity did not reveal group-specific community structure. Optimal solutions suggested either four or two clusters based on average silhouette width, and we found that the distribution of participants across clusters did not differ by PMOS status (p=1.00). These findings further support the absence of major differences in overall gut microbiome community structure associated with PMOS.

### 3.3 Indicator species analysis and differential abundance assessment

Although global diversity and overall community structure did not differ between groups, these ecological metrics may obscure taxa that are preferentially associated with specific clinical categories. Indicator species analysis (ISA) identifies taxa that are both frequent within a group (high fidelity) and relatively exclusive to that group (high specificity), combining these components into an indicator value (IndVal) ranging from 0 to 1. We therefore performed ISA at the species level after applying prevalence and abundance filtering at the ASV level prior to agglomeration.

**Figure 3:**
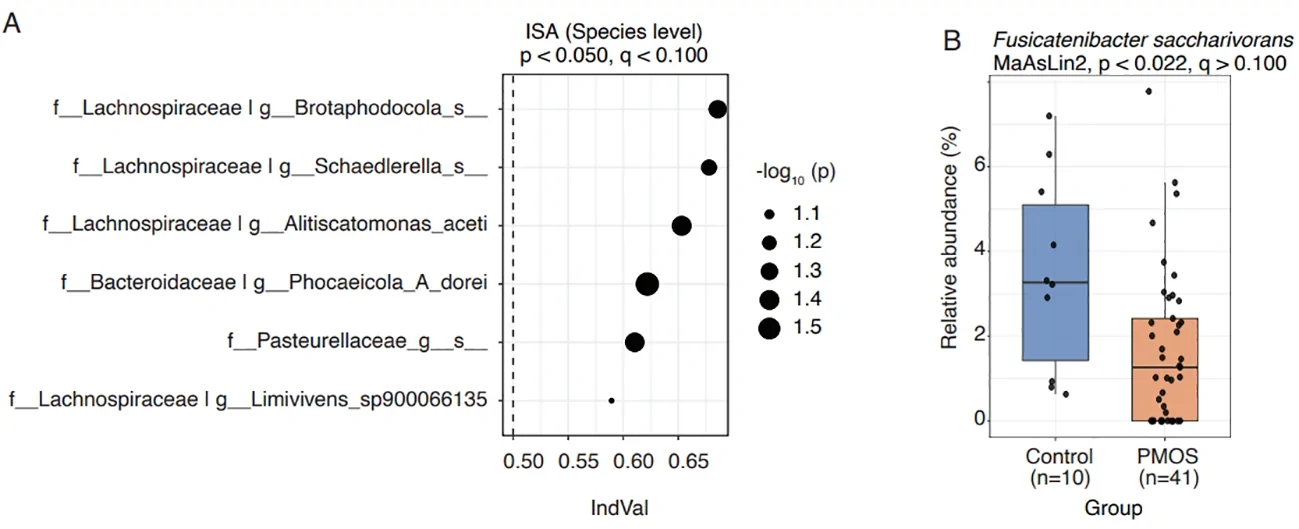
Species-level indicator analysis and differential abundance. **(A)** Indicator species analysis (ISA) showing species associated with the control group after FDR correction (p<0.05, q<0.10). Dot size represents −log_10_(p-value), and the dashed line indicates IndVal=0.5. **(B)** Relative abundance of *Fusicatenibacter saccharivorans* based on MaAsLin2.

In the full cohort (n=51), six species were significantly associated with the control group after false discovery rate correction (q<0.10). These included *Phocaeicola_A dorei* (IndVal=0.62, p=0.026), *Alitiscatomonas aceti* (IndVal=0.65, p=0.039), an unclassified Pasteurellaceae taxon (IndVal=0.61, p=0.041), *Brotaphodocola* (IndVal=0.69, p=0.046), *Schaedlerella* (IndVal=0.68, p=0.056), and *Limivivens* (IndVal=0.59, p=0.085, **Figure 3A**). All taxa were more prevalent among controls than PMOS participants. For example, *Brotaphodocola* and *Schaedlerella* were detected in 70% of controls compared with 34.1% and 36.6% of PMOS participants, respectively. Similarly, *Alitiscatomonas aceti* was present in 60% of controls versus 24.4% of PMOS participants, and *Phocaeicola_A dorei* in 50% versus 14.6%, respectively. These differences in prevalence support the observed specificity of these taxa to the control group.

In the sensitivity analysis excluding participants with recent antibiotic use (n=36; 8 controls and 28 PMOS), two species remained nominally associated with the control group: *Brotaphodocola* (IndVal=0.74, p=0.024) and *Schaedlerella* (IndVal=0.72, p=0.045). In this subset, both taxa were present in 75% of controls compared with 28.6% and 32.1% of PMOS participants, respectively. Although these associations did not survive FDR correction in the reduced sample, the persistence of large prevalence differences suggests that the control-associated signal is not driven by recent antibiotic exposure. No species were identified as indicators of the PMOS group in either analysis. Collectively, these exploratory findings suggest that PMOS may be associated with reduced prevalence of certain commensal taxa, particularly members of the order Lachnospirales; however, these observations require validation in larger cohorts.

Multivariable differential abundance analyses using MaAsLin2 did not identify taxa significantly associated with PMOS after correction for multiple testing. At nominal significance levels, *Fusicatenibacter saccharivorans* was reduced in PMOS participants, although this association did not remain significant after false discovery rate adjustment in analyses including and excluding antibiotics (**Figure 3B**). This was consistent across all taxonomic levels evaluated, and no taxa reached the predefined false discovery rate threshold. These findings indicate that PMOS status was not associated with robust, taxon-specific differences in gut microbiome composition in this cohort.

### 3.4 Gut microbiome and metabolic dysfunction

Importantly, the PMOS and control groups in this cohort did not differ substantially in BMI or metabolic markers, which may partially explain the limited microbiome separation observed between groups. Because PMOS is frequently accompanied by metabolic dysfunction, we assessed whether gut microbial diversity correlated with markers of insulin resistance and glycemic status. Spearman correlation analysis revealed no significant associations between any alpha diversity metrics and metabolic markers, including HOMA-IR, fasting insulin, HbA1c, or BMI (all p>0.320, q>0.963, **Supplementary Table 4**). Collectively, these findings indicate that PMOS status was not associated with differences in overall gut microbial alpha diversity in this cohort, regardless of metabolic profile or recent antibiotic exposure. In PERMANOVA models adjusted for age, BMI, and PMOS status, metabolic variables were not significantly associated with overall community composition across Aitchison, Bray–Curtis, or Jaccard distances (all p≥0.175, **Supplementary Table 3**). The proportion of variance explained by these markers was minimal (R²=0.011–0.024), comparable to or lower than that explained by PMOS status alone (**Supplementary Table 3**). These findings suggest that insulin resistance and related metabolic measures were not strongly associated with gut microbiome structure within this cohort, although modest associations may have been undetectable due to limited sample size and inter-individual variability. Although overall community composition did not differ.

### 3.5 Gut microbiome assessment in regard to circulating androgen levels and reproductive characteristics

We further assessed whether circulating androgen concentrations were associated with gut microbiome diversity. No significant correlations were observed between alpha diversity metrics and total testosterone, FAI, or SHBG (Spearman’s ρ range−0.10 to 0.16; all p≥0.276; all q=0.964, **Supplementary Table 4**). PERMANOVA analyses adjusted for age, BMI, and PMOS status likewise showed no significant associations between androgen levels and overall microbial composition across Bray–Curtis, Aitchison, or Jaccard distances. Although marginal trends were observed for total testosterone and FAI using Jaccard distance (p≈0.095), the variance explained remained small (R²≈0.023, **Supplementary Table 3**). Tests for homogeneity of dispersion were not significant, and ordination plots did not reveal clustering by androgen levels (**Supplementary Table 3**). Collectively, these results suggest that circulating androgens do not substantially shape global gut microbiome structure in this cohort.

**Figure 4:**
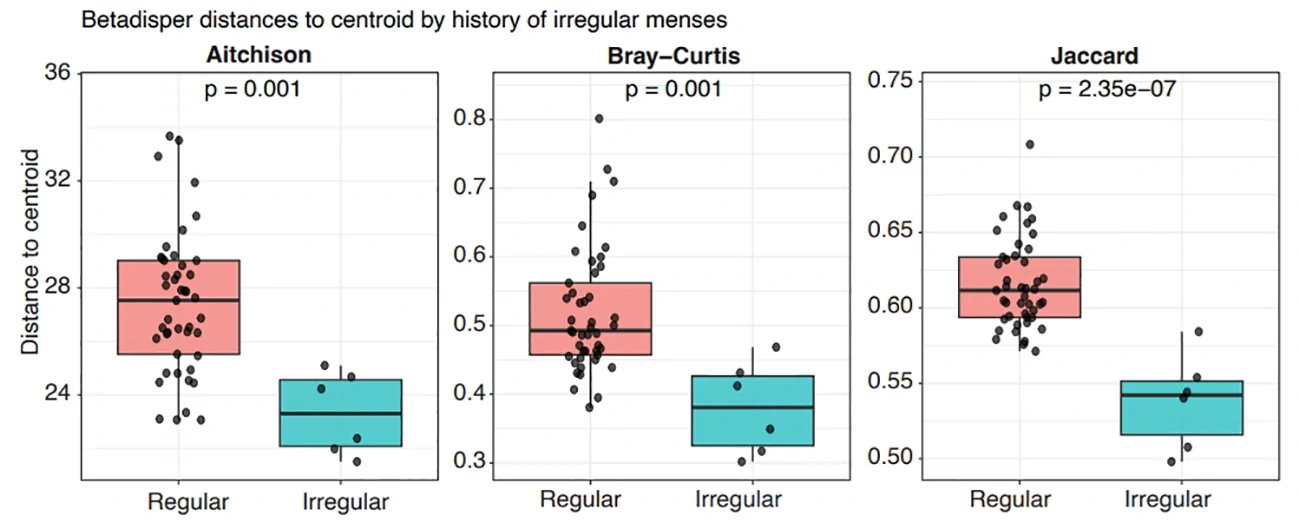
Beta dispersion analysis of gut microbiome composition according to history of irregular menses. Distance to centroid (*betadisper*) was calculated for Aitchison, Bray–Curtis, and Jaccard distances. Boxplots show the distribution of distances to the group centroid for women with regular and irregular menses history, with individual samples shown as points. P-values correspond to tests for homogeneity of multivariate dispersion between groups.

Finally, we evaluated whether menstrual and reproductive features were associated with gut microbiome diversity. No significant differences in alpha diversity were observed across variables, including history of irregular menses, current oligomenorrhea, amenorrhea, difficulty getting pregnant, menstrual length, or regular cycle status (all q>0.90). Similarly, PERMANOVA models adjusted for age and BMI showed no significant associations between these features and overall microbial composition across Aitchison, Bray–Curtis, or Jaccard distances (all p>0.16), with very small proportions of explained variance (R²<0.025, **Supplementary Table 3**). However, analysis of multivariate homogeneity of dispersion revealed that a history of irregular menses was associated with significantly greater within-group variability across all distance metrics (Bray–Curtis p=0.0007; Jaccard p<0.0001; Aitchison p=0.0005, **Supplementary Table 3**; **Figure 4**). No other reproductive variable showed significant dispersion differences. These findings suggest that while menstrual irregularity is associated with increased microbiome heterogeneity, reproductive features do not consistently drive shifts in overall community composition.

## 4 Discussion

In this study, we assessed the gut microbiome in women with PMOS, integrating taxonomic and clinical association analyses. Contrary to prior literature, our findings demonstrate that PMOS status was not associated with differences in global gut microbial diversity or overall community structure, even after accounting for key confounders, including age, BMI, and recent antibiotic exposure. Instead, our results suggest that host-related factors and microbial heterogeneity, rather than disease status, may play a more prominent role in shaping gut microbiome variation in this cohort. Nonetheless we acknowledge the relatively small control group (n=10) with the 41 cases, pointing a limited statistical power for detecting subtle microbiome differences. Nonetheless, we began evaluating the influence of PMOS-specific medications—such as metformin, oral contraceptives (OCPs), and GLP-1 agonists—to account for potential confounding effects, though the primary microbial comparisons remained non-significant. Specifically, we observed no significant differences in alpha or beta diversity between PMOS and control women. These findings contrast with several prior studies reporting reduced diversity or altered community structure in PMOS (32, 33), but are consistent with an emerging body of literature indicating substantial heterogeneity and limited reproducibility across PMOS microbiome studies (34, 35). Differences in study design, population characteristics, sequencing methodologies, and confounder adjustment likely contribute to these inconsistencies. Emerging evidence suggests that host factors such as age and BMI are stronger determinants of gut microbiome composition than PMOS diagnosis. Studies controlling for BMI have shown minimal differences in microbial diversity and overall composition between PMOS and control groups, suggesting that obesity and metabolic status may be primary drivers of microbiome variation rather than PMOS (36). Furthermore, within our PMOS cohort, variance partitioning analyses revealed that BMI and age explained a greater proportion of microbial variation than PMOS status, reinforcing the concept that metabolic and demographic factors may overshadow disease-specific microbial signatures. This aligns with prior evidence suggesting that obesity-related metabolic disturbances are major drivers of microbiome composition (37). Additionally, while hyperandrogenism and metabolic abnormalities are central features of PMOS, their direct influence on microbiome composition appears modest and inconsistent across studies (11, 38). These findings suggest that, within this cohort, metabolic dysfunction and androgen excess do not exert strong, detectable effects on global microbiome structure. Microbiome–host interactions in PMOS may occur at a more functional or strain-specific level, rather than through broad compositional changes.

Although overall community composition did not differ significantly between groups, Jaccard dispersion analyses indicated greater variability in microbial presence/absence patterns among PMOS participants. These findings suggest increased inter-individual heterogeneity rather than a consistent disease-specific microbial signature. Systematic evidence shows that while reductions in alpha diversity are observed in some cohorts, overall community composition remains highly variable, underscoring the absence of a consistent microbial signature in PMOS (39). This variability likely reflects differences in host metabolic status, including obesity and insulin resistance, as well as environmental exposures, dietary patterns, and population-specific characteristics, all of which are known to shape the gut microbiome. Furthermore, accumulating evidence suggests that the relationship between the gut microbiome and PMOS is complex and not necessarily causal, supporting a multifactorial and context-dependent framework rather than a uniform disease-specific profile (11, 40). A similar pattern emerged in analyses of reproductive traits, where a history of irregular menses was strongly associated with increased microbiome dispersion, consistent with the broader variability reported across PMOS microbiome studies (39). These findings are consistent with ecological models of dysbiosis that emphasize loss of stability and increased inter-individual variability rather than uniform compositional shifts (41). The association between irregular menses and increased microbiome dispersion—rather than a shift in mean composition—may reflect hormonal *unpredictability* rather than a single altered hormonal state. Regularly cycling women experience a consistent, rhythmic pattern of estrogen and progesterone fluctuation, which shapes gut motility, bile acid metabolism, and mucosal immunity via the estrobolome, the subset of gut bacteria capable of deconjugating estrogens through β-glucuronidase activity (42). In irregular or anovulatory cycles, this rhythmicity is disrupted, producing a more variable hormonal environment that differs unpredictably between individuals rather than converging on a distinct profile. Such variability would be expected to manifest as increased inter-individual heterogeneity (dispersion) rather than a shared compositional signature detectable by PERMANOVA—consistent with ecological models in which loss of physiological stability first appears as reduced community resilience before any consistent directional (41). Irregular menses also often co-occurs with other sources of physiological variability, including inconsistent ovulatory hormone surges, variable insulin resistance, and fluctuating stress/cortisol exposure, each independently linked to microbiome instability (43). Menstrual irregularity may thus serve as a clinical proxy for underlying hormonal and metabolic instability that destabilizes the gut microbiome at the level of individual variability, rather than driving a convergent, PMOS-specific signature. Consistent with the lack of global compositional differences, multivariable differential abundance analysis did not identify any taxa significantly associated with PMOS after correction for multiple testing. This underscores the limited robustness of disease-specific microbial biomarkers in this cohort. However, indicator species analysis revealed that several taxa, including *Phocaeicola dorei*, *Alitiscatomonas aceti*, *Schaedlerella*, and *Brotaphodocola*, were preferentially associated with the control group. Notably, many of these taxa belong to commensal groups within the order Lachnospirales, which are often linked to SCFA production and gut health. Their reduced prevalence in PMOS participants suggests that PMOS may be characterized by the depletion of beneficial commensals rather than by the enrichment of pathogenic taxa. Notably, several PMOS studies have reported depletion of beneficial SCFA–producing taxa, such as members of *Lachnospiraceae*, supporting said hypothesis (39). This pattern is further supported by the nominal reduction of *Fusicatenibacter saccharivorans* in PMOS, a species associated with anti-inflammatory properties and metabolic health. Although these associations did not remain significant after multiple-testing correction, the consistent directionality and prevalence differences suggest subtle ecological shifts not captured by traditional differential abundance approaches. It is also important to discuss the apparent differences between reports of, for example *Phocaeicola* species acting pathogenically in PCOS in other studies (44) and evidence for a protective role of *P. dorei* (former *Bacteroides fragilis*) such as in our study, likely reflects genuine strain- and species-level heterogeneity rather than a fixed pro- or anti-inflammatory identity. A recent study showed that *P. dorei* and its close relative *P. vulgatus* differ substantially in genome plasticity, mobile element content, anti-phage defenses, and carbohydrate-active enzyme profiles despite their taxonomic closeness, meaning abundance alone can obscure important functional differences *(*45*)*. Consistent with this, another study found that in cholestatic liver fibrosis, native *P.* abundance rises with disease severity. Still, this expansion reflects a functionally eroded variant that has lost key symbiotic gene modules (e.g., SusC/SusD polysaccharide utilization loci). In contrast, a therapeutic strain with intact symbiotic architecture was antifibrotic and immunomodulatory when administered exogenously, demonstrating that abundance and function can decouple (46). Together, these findings suggest that pathogenic associations reported for *Phocaeicola* in PMOS/PCOS should not be generalized to the species level especially in these low sample number and short read data results; future work should characterize strain-specific genomic and functional attributes to clarify whether particular *P. dorei* strains are protective or pathoadaptive in this context.

Despite the well-established links between PMOS, insulin resistance, and hyperandrogenism, we found no significant associations between gut microbiome diversity and metabolic markers or androgen levels. Also, in a previous study on PMOS, 16S rRNA sequencing of the V3-V4 region was employed, and a higher abundance of *Streptococcus* was found in the intestines of PMOS patients than in healthy individuals. In addition, this study revealed that androgen levels and BMI were significantly increased in PMOS patients with elevated *Streptococcus* abundance (32). These findings suggest that *Streptococcus* may be involved in PMOS development. On the other hand, testosterone has also been associated with gut dysbiosis. In laboratory studies of testosterone exposure, the relative abundance of bacteria involved in steroid production (*Nocardiaceae* and *Clostridiaceae*) and the production of unsaturated SCFAs increases.

Collectively, our findings do not support the presence of a robust, disease-specific gut microbiome signature associated with PMOS in this cohort. Instead, the data suggest subtle ecological differences characterized by increased inter-individual variability and possible depletion of select commensal taxa. Host factors, including BMI, age, and reproductive characteristics, play a dominant role in shaping microbiome composition. These findings highlight the importance of moving beyond traditional diversity and taxonomic analyses toward multi-omics and functional approaches to better understand microbiome contributions to PMOS. Because this study used 16S rRNA gene sequencing, functional interpretations regarding SCFA production, inflammatory signaling, or microbial metabolic activity remain speculative and should be validated using metagenomic, metatranscriptomic, or metabolomic approaches. Key strengths, however, include the use of multivariable-adjusted models, multiple complementary diversity metrics, and sensitivity analyses excluding antibiotic users. Additionally, integrating ecological and taxonomic approaches provides a comprehensive assessment. However, limitations include a small sample size, particularly in the control group, which reduces statistical power. The cross-sectional design precludes causal inference, and the use of 16S rRNA sequencing limits functional resolution. Unmeasured confounders such as diet and lifestyle may also influence results. Additionally, the control group had significantly greater parity and pregnancy history than the PMOS group, and this imbalance was not adjusted for in our models. Pregnancy induces substantial and lasting changes in host physiology—including shifts in sex steroid and metabolic hormone exposure, immune tolerance, and gut barrier function—that have been shown to produce long-term alterations in gut microbiome composition persisting years after delivery. It is therefore possible that the greater parity in the control group contributed to some of the observed microbial heterogeneity or to the modest taxonomic differences identified between groups, independent of PMOS status itself. Future studies should match groups on parity and pregnancy history, or explicitly adjust for these variables, to disentangle their contribution from PMOS-specific effects. In conclusion, this study did not identify major alterations in global gut microbiome diversity or composition associated with PMOS in Puerto Rican women. However, exploratory analyses suggest increased microbial heterogeneity and reduced prevalence of select commensal taxa. Larger longitudinal studies incorporating functional multi-omics approaches are needed to clarify the role of the gut microbiome in PMOS pathophysiology (47).

## Data Availability

The datasets presented in this study can be found in online repositories. The names of the repository/repositories and accession number(s) can be found below: https://qiita.ucsd.edu/study/description/12884, QIITA. Additionally, sequence data has been deposited in the European Nucleotide Archive Study: ERP202883, https://www.ebi.ac.uk/ena/browser/view/ERP202883.
